# Supplementary material for: Altered Ca2+ Homeostasis in Red Blood Cells of Polycythemia Vera Patients Following Disturbed Organelle Sorting during Terminal Erythropoiesis
Source: Cells. 2021 Dec 24;11(1):49. doi: 10.3390/cells11010049 (PMC8750512; doi:10.3390/cells11010049)
Supplement: Supplementary file 1 [file cells-11-00049-s001.zip › Buks et al Supplemental Material.pdf]

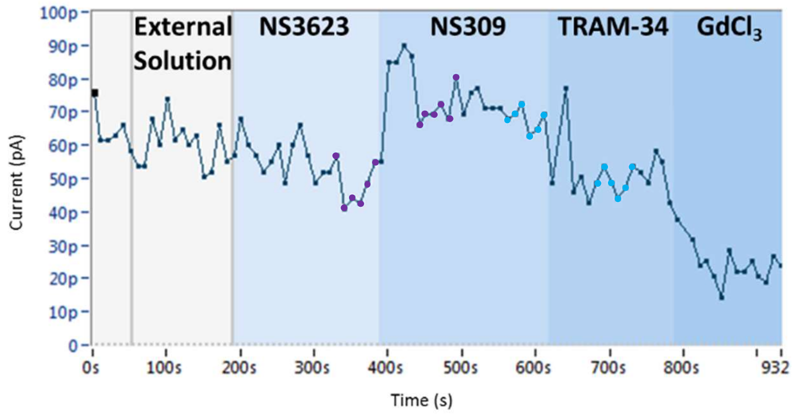

Supplemental Figure S1. Supplemental Figure S1. Patch-clamp protocol timeline with a representative RBC response from a healthy individual. Upon cell catch external solution was added to the wells followed by 10 μM NS3623, 10 μM NS309, 5 μM TRAM-34 and 30 μM GdCl3. Currents were measured at room temperature applying -100 to +80 mV ramp voltage protocol for 300 ms, at a holding potential of -30 mV. The cell response was measured in pA at +80 mV.

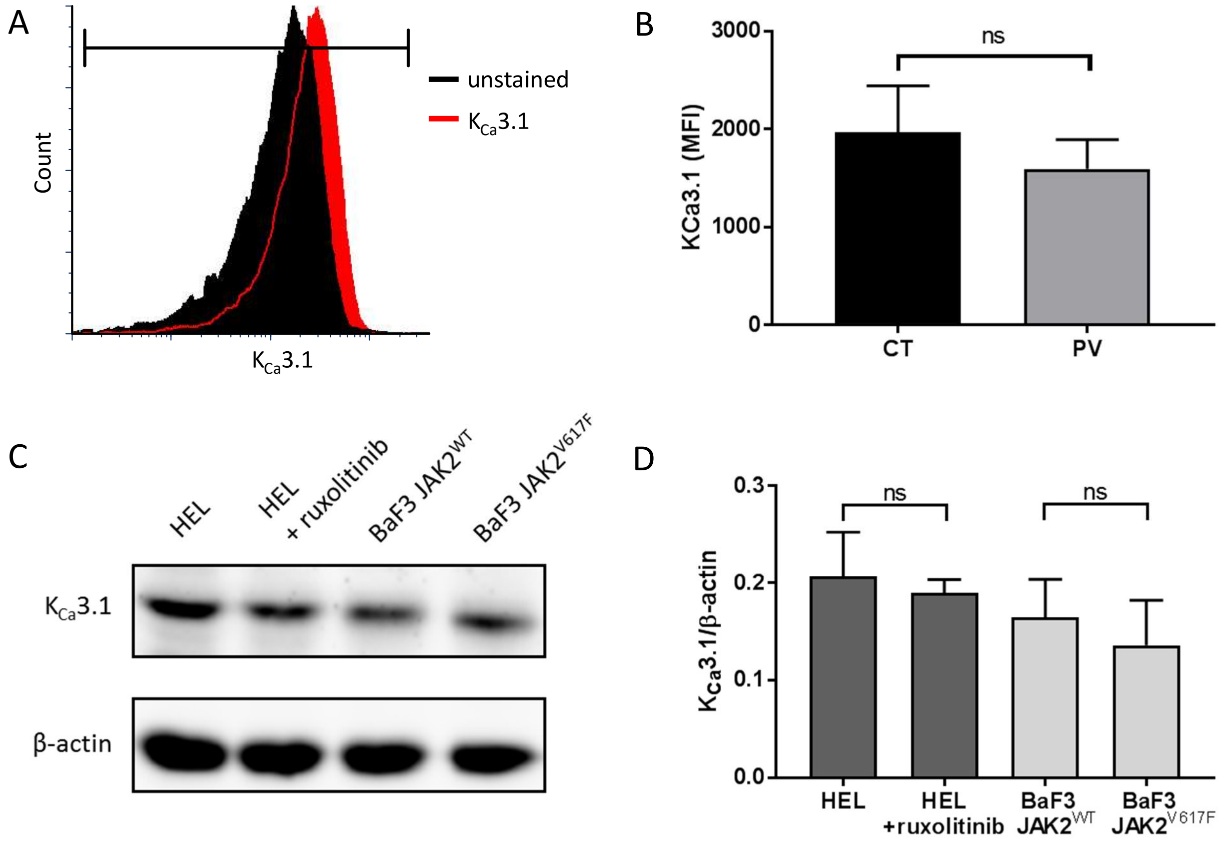

Supplemental Figure S2. JAK2^{V617F} does not alter Gárdos expression in PV RBCs, BaF3 JAK2^{V617F} and HEL cell lines. Representative flow cytometry histograms of $K_{Ca}3.1$ expression on **A)** CT RBC surface (red), unstained RBCs (black). **B)** $K_{Ca}3.1$ MFI quantification in CT (black) and PV (grey) RBCs; N=4, Mann-Whitney test, ns – not significant. **C)** Representative SDS-PAGE immunoblot of $K_{Ca}3.1$ expression in HEL with or without 0.3 μM ruxolitinib treatment for 24h and BaF3 JAK2^{WT}, and BaF3 JAK2^{V617F}. **D)** Quantitative analysis of $K_{Ca}3.1$ expression from SDS-PAGE immunoblots in HEL cells with or without 0.3 μM ruxolitinib treatment for 24h and BaF3 JAK2^{WT}, and BaF3 JAK2^{V617F} cells; N=4, Mann-Whitney test, ns – not significant.

Supplemental Table S1. Quality control filters. Only NS309 and TRAM-34 responder cells were used in the analysis. A cell was considered NS309 responder, if it displayed at least 20% current increase compared to baseline. For RBCs additional QC of at least 10 pA increase was applied to avoid false-positive signal from noise. NS309 induced currents over 500 pA for BaF3 cells and over 400 pA HEL cells were considered as outgroups. No such outgroups were observed in RBCs. A cell was considered TRAM-34 responder, if it displayed at least 20% current decrease compared to the current upon NS309 addition. For RBCs additional QC of at least 5 pA decrease was applied to avoid false-positive signal from noise. Inf – infinity.

	Quality Control filters	RBCs		BaF3		HEL	
		Lower value	Higher value	Lower value	Higher value	Lower value	Higher value
Seal resistance (MΩ)	Cell catch	5	Inf	5	Inf	5	Inf
	Whole-cell	50	Inf	50	Inf	50	Inf
	Before compound addition	900	Inf	500	Inf	500	Inf
	End of the experiment	1500	Inf	500	Inf	500	Inf
Current (pA)	Inward current values	-Inf	-20	-Inf	-25	-Inf	-25
	Outward current values	16	Inf	18	Inf	18	Inf
	NS309 responders	20 %, ∆10	Inf	20 %	500	20 %	400
	TRAM-34 block	20 %, ∆5	Inf	20 %	Inf	20 %	Inf
