# Supplementary material for: Altered Ca2+ Homeostasis in Red Blood Cells of Polycythemia Vera Patients Following Disturbed Organelle Sorting during Terminal Erythropoiesis
Source: Cells. 2021 Dec 24;11(1):49. doi: 10.3390/cells11010049 (PMC8750512; doi:10.3390/cells11010049)
Supplement: Supplementary file 1 [file cells-11-00049-s001.zip › Supplemental Material captions.pdf]

Supplemental Table S2.

Table S2 Perseus analysis.xlsx. Whole cell proteome was analyzed using nanoscale liquid chromatography coupled to tandem mass spectrometry (MS/MS). Raw data were exported from the Maxquant software and analyzed in Perseus software.

Supplemental Table S3.

Table S3 Perseus analysis by groups.xlsx. Whole cell proteome was analyzed using nanoscale liquid chromatography coupled to tandem mass spectrometry (MS/MS). Raw data were exported from the Maxquant software and analyzed in Perseus software. The appendix is an optional section that can contain details and data supplemental to the main text—for example, explanations of experimental details that would disrupt the flow of the main text but nonetheless remain crucial to understanding and reproducing the research shown.

Supplemental Table S4.

Table S4 On off proteins.xlsx. Table of on/off proteins in red blood cells and reticulocytes from health individuals and polycythemia vera patients. On/off protein was defined as a protein detected in at least three samples out of four per group one, and no more than one sample out of four in group two. Healthy individuals (CT), Polycythemia Vera (PV), and red blood cells (RBC).
